# Phenotypic plasticity, but not adaptive tracking, underlies seasonal variation in post‐cold hardening freeze tolerance of *Drosophila melanogaster*

**DOI:** 10.1002/ece3.5887

**Published:** 2019-12-06

**Authors:** Helen M. Stone, Priscilla A. Erickson, Alan O. Bergland

**Affiliations:** ^1^ University of Virginia Charlottesville Virginia

**Keywords:** cold hardening, *Drosophila melanogaster*, freeze tolerance, plasticity, seasonal evolution

## Abstract

In temperate regions, an organism's ability to rapidly adapt to seasonally varying environments is essential for its survival. In response to seasonal changes in selection pressure caused by variation in temperature, humidity, and food availability, some organisms exhibit plastic changes in phenotype. In other cases, seasonal variation in selection pressure can rapidly increase the frequency of genotypes that offer survival or reproductive advantages under the current conditions. Little is known about the relative influences of plastic and genetic changes in short‐lived organisms experiencing seasonal environmental fluctuations. Cold hardening is a seasonally relevant plastic response in which exposure to cool, but nonlethal, temperatures significantly increases the organism's ability to later survive at freezing temperatures. In the present study, we demonstrate seasonal variation in cold hardening in *Drosophila melanogaster* and test the extent to which plasticity and adaptive tracking underlie that seasonal variation. We measured the post‐cold hardening freeze tolerance of flies from outdoor mesocosms over the summer, fall, and winter. We bred outdoor mesocosm‐caught flies for two generations in the laboratory and matched each outdoor cohort to an indoor control cohort of similar genetic background. We cold hardened all flies under controlled laboratory conditions and then measured their post‐cold hardening freeze tolerance. Comparing indoor and field‐caught flies and their laboratory‐reared G1 and G2 progeny allowed us to determine the roles of seasonal environmental plasticity, parental effects, and genetic changes on cold hardening. We also tested the relationship between cold hardening and other factors, including age, developmental density, food substrate, presence of antimicrobials, and supplementation with live yeast. We found strong plastic responses to a variety of field‐ and laboratory‐based environmental effects, but no evidence of seasonally varying parental or genetic effects on cold hardening. We therefore conclude that seasonal variation in post‐cold hardening freeze tolerance results from environmental influences and not genetic changes.

## Graphical abstract

Post‐cold hardening freeze tolerance varies seasonally in *Drosophila melanogaster* reared in outdoor mesocosms. This temporal variation is a product of plasticity and not adaptive tracking, as the seasonal changes are not heritable in G1 and G2 offspring. Cold hardening is influenced by a variety of factors, including the thermal environment, nutrition, age, and larval density.

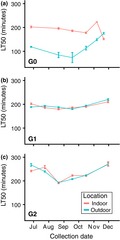

## 1 INTRODUCTION

All organisms residing in temperate climates must cope with seasonal fluctuations in their environment. Many species exhibit phenotypic plasticity, which grants them the flexibility to thrive during the growing season and survive unfavorable times. For example, aspects of cold tolerance are known to vary as a function of seasonal exposure and provide a mechanism for some species to successfully overwinter (Anderson, Chevone, & Hess, 1992; Esterbauer & Grill, 1978; Shearer et al., 2016). While phenotypic variation can arise as a result of environmentally triggered plasticity, genetic variation in seasonally advantageous traits also exists (Dobzhansky & Ayala, 1973; reviewed in Tauber & Tauber, 1981 and Williams et al., 2017). Therefore, genotypes that underlie variation in seasonally relevant phenotypes may change in frequency across seasonal timescales for short‐lived organisms (Behrman, Watson, O'Brien, Heschel, & Schmidt, 2015; Grosberg, 1988; Hazel, 2002; King, 1972; Schmidt & Conde, 2006). In the present study, we examine the relative importance of plasticity and rapid, seasonal adaptation in the cold tolerance of *Drosophila melanogaster*.

*Drosophila melanogaster* is an ideal system for contrasting the importance of phenotypic plasticity and rapid adaptation as mechanisms for survival under seasonally fluctuating conditions. Notably, *D. melanogaster* has a short generation time, producing 10–15 generations per growing season (Pool, 2015), and experiences dramatic changes in selection pressures across seasons that elicit rapid adaptation in life‐history traits (Behrman et al., 2015). Populations of flies living in orchards evolve over the period of months (Bergland, Behrman, O'Brien, Schmidt, & Petrov, 2014) as they track changing fitness optima influenced by seasonal fluctuations in selection pressure (Machado et al., 2018). Although many life‐history and stress tolerance traits have been shown to exhibit adaptive tracking across short timescales (e.g., Behrman et al., 2018; Behrman et al., 2015; Cogni et al., 2014, 2015), some of these traits are also highly plastic (e.g., Ayrinhac et al., 2004; Chippindale, Leroi, Kim, & Rose, 2004). In general, when environmental pressures vary over timescales briefer than the lifespan of the organism, plasticity—as opposed to adaptive tracking—is more likely to occur (Botero, Weissing, Wright, & Rubenstein, 2015; Levins, 1968). For instance, physiological responses to temperature may be more likely to exhibit plasticity because temperature can change rapidly over short timescales. In the present study, we examine the roles of plasticity and rapid adaptation in seasonally varying phenotypes using cold hardening in *D. melanogaster*.

Many species of insects plastically adapt to cold seasons via cold hardening, a phenomenon in which brief pre‐exposure to cool temperatures results in greater cold tolerance (Chen, Denlinger, & Lee, 1987; Lee, Chen, & Denlinger, 1987; Lee et al., 2006). *Drosophila melanogaster* is capable of cold hardening, with an increase in cold tolerance resulting from pre‐exposure periods as brief as half an hour (Czajka & Lee, 1990). Cold hardening has been documented in larvae, pupae, and adults (Jensen, Overgaard, & Sørensen, 2007; Koštál et al., 2011; Rajamohan & Sinclair, 2008). Cold hardening in flies is associated with widespread transcriptional changes (MacMillan et al., 2016; Qin, Neal, Robertson, Westwood, & Walker, 2005), shifts in metabolite profiles (Overgaard et al., 2007), and altered lipid composition of cellular membranes (Lee et al., 2006; Overgaard, Sørensen, Petersen, Loeschcke, & Holmstrup, 2006). Collectively, these responses permit maintenance of neuronal homeostasis under cold stress (Armstrong, Rodríguez, & Robertson, 2012) and reduce apoptosis following cold injury (Yi, Moore, & Lee, 2007). However, cold hardening also carries costs in terms of future reproductive output (Everman, Delzeit, Hunter, Gleason, & Morgan, 2018; Overgaard et al., 2007), suggesting that avoiding cold hardening could be beneficial if temperatures never drop to lethally cold. Individual *D. melanogaster* genotypes vary in their cold hardening response (Gerken, Eller, Hahn, & Morgan, 2015; Gerken, Eller‐Smith, & Morgan, 2018); therefore, different cold hardening abilities may be advantageous under different conditions. In addition to genetic influences, specific aspects of environmental exposure, such as the temperature and duration of cold exposure (Czajka & Lee, 1990; Kelty & Lee, 1999), also affect cold hardening. We thus hypothesized that the cold hardening response might vary seasonally in temperate climates via some combination of genetic adaptation and environmental influences.

In the present study, we ask whether the post‐cold hardening freeze tolerance of *D. melanogaster* varies seasonally in field‐reared populations, and if so, whether this variation occurs as a result of environmental influences, genetic changes, or some combination thereof. Although other studies have examined the occurrence of rapid cold hardening in the field (e.g., Kelty, 2007; Overgaard & Sørensen, 2008), in this study we measure the effect of exposure to natural seasonal conditions on freeze survival following a consistent cold hardening treatment. Since sample size limitations prevented us from assaying the baseline freeze tolerance of the flies in our experiment, we can only effectively measure post‐cold hardening freeze tolerance rather than the ability to cold harden itself. While flies can cold harden over a timescale of hours, we used a 2‐week cold hardening protocol in order to emulate seasonal, rather than rapid, cold hardening; seasonal cold hardening is most protective for overwintering and so was more fitting for our study (Teets & Denlinger, 2013). Over the course of multiple seasons in a single year, we collected flies from outdoor mesocosms and then subjected them to a controlled pre‐exposure to cool temperatures (cold hardening treatment) followed by a freeze tolerance test. By contrasting outdoor‐caught flies (G0), their laboratory‐reared offspring (G1), and their grandchildren (G2) to similarly treated flies that were reared entirely indoors, we tested the impact of seasonally varying environmental, parental, and genetic effects on cold hardening. We found that post‐cold hardening freeze tolerance does not evolve over seasonal timescales but shows dramatic season‐specific plasticity. We further show that this plasticity is potentially caused by thermal exposure in the field. Using additional laboratory experiments, we identified other possibly influential factors besides temperature, such as nutrition, age, and larval density. Taken together, our work suggests that cold hardening is a highly plastic trait that does not exhibit classic signatures of adaptive tracking.

## 2 METHODS

**Figure 1:**
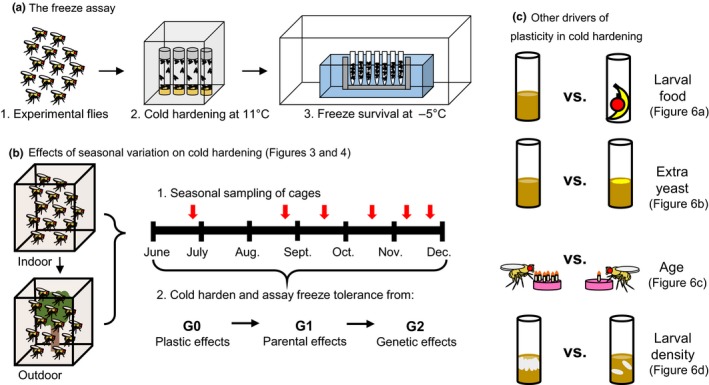
Overview of experimental methods. (a) Flies were subjected to various experimental or field conditions to test the role of genetics and environment on post‐cold hardening freeze tolerance. Flies were then placed in cold hardening chambers at 11°C for 2 weeks. After cold hardening, freeze tolerance was assessed by placing flies at −5°C for varying amounts of time to establish survival curves. (b) The effects of seasonal variation on post‐cold hardening freeze tolerance were tested by sampling related indoor and outdoor fly populations across the seasons. The indoor flies served as a control, while the outdoor flies experienced varying seasonal conditions. In the laboratory, sampled flies were bred to the G1 and G2 generations. The post‐cold hardening freeze tolerance of each generation of indoor and outdoor flies was measured in order to elucidate seasonally varying plastic effects (G0), parental effects (G1), and genetic effects (G2) on post‐cold hardening freeze tolerance. (c) We assessed other potential drivers of plasticity in post‐cold hardening freeze tolerance using laboratory experiments. Flies from various experimental conditions, including flies fed different foods during development, flies supplemented with extra yeast, flies of different ages, and flies reared at different larval densities, were cold hardened and tested for freeze tolerance

Our study examined seasonal variation in freeze survival following extended cold hardening and elucidated whether such variation originated from phenotypic plasticity or genetic adaptive tracking. We quantified cold tolerance by subjecting flies to a cold hardening treatment followed by a freeze tolerance test (Figure 1a). Over the course of one sampling season, we collected flies of similar genetic backgrounds from indoor and outdoor mesocosms and measured their post‐cold hardening freeze tolerance to determine how flies exposed to a seasonally fluctuating environment may cold harden differently from indoor controls (Figure 1b). In the laboratory, we bred indoor‐ and outdoor‐caught flies for two generations. We assayed the indoor and outdoor cage flies' laboratory‐reared offspring (G1 generation), raised in a common environment, to test for parental effects on cold hardening. In turn, we tested the post‐cold hardening freeze tolerance of the second laboratory‐reared generation (G2). The G2 flies were now stripped of any field‐induced environmental differences and parental effects; assaying these flies tested for evidence of adaptive tracking occurring in the seasonally exposed population as compared to the environmentally stable control.

The indoor and outdoor cages likely experienced varying conditions aside from the thermal environment. In order to test the impact of these other potential drivers of plasticity, we conducted cold hardening and freeze assays on indoor flies fed different diets, indoor flies of different ages, and indoor flies reared at varying larval densities (Figure 1c).

### 2.1 The hybrid swarm

Using inbred fly lines from various regions of the Eastern United States and the Bahamas, we created two outbred and genetically diverse populations of flies (populations A and B) for use in our experiments. We created each population by crossing a total of 34 fly lines representing each of our chosen regions, with the particular lines from each region picked at random. We used flies from Bowdoinham, Maine (NCBI BioProject # PRJNA383555); Ithaca, New York (Grenier et al., 2015); spring and fall collections from Linvilla Orchard, Media, Pennsylvania (Behrman et al., 2018); Raleigh, North Carolina (Mackay et al., 2012); the Southeastern United States (Kao, Zubair, Salomon, Nuzhdin, & Campo, 2015), and the Bahamas (Kao et al., 2015). The initial crosses were established with four sets of 34 round‐robin crosses. After each population was established, we maintained them with 2‐week, nonoverlapping generations in indoor mesh cages (2 m × 2 m × 2 m; Bioquip product number 1406C) at a population size of approximately 10,000 flies per generation. Each generation received approximately 5 L of standard cornmeal–molasses media sprinkled with live baker's yeast. We reared the flies in the laboratory for approximately 32 generations before transferring subsets of each population to outdoor cages in June 2018.

### 2.2 Cold hardening

We cold hardened flies by placing them in a temperature‐controlled chamber at approximately 11°C with a 9L:15D photoperiod for 13–15 days (typically 14 days; longer or shorter cold hardening periods occasionally occurred). During cold hardening, we held flies in vials containing cornmeal–molasses food. Vials contained 25 male flies each except in rare circumstances when fewer flies were available. We sorted flies using CO_2_ sedation. We sedated flies for a maximum of 20 min, which is well under the sedation duration found to impact the cold hardening response (Nilson, Sinclair, & Roberts, 2006).

### 2.3 The freeze assay

We tested the freeze tolerance of flies by subjecting them to −5°C temperatures. We froze subsets of flies for varying spans of time, ranging from approximately 2–5 hr, in order to generate a freeze survival curve.

Following cold hardening, we noted the number of flies that had died during the cold hardening process prior to conducting the freeze assay. We transferred each vial of flies to a 5‐mL snap‐cap Falcon tube and suspended the tubes in a salt water solution (~3 M NaCl) held at approximately −5°C within a chest freezer. We used weighted blocks to keep the tubes submerged in liquid up to the rim of the cap. In order to minimize temperature fluctuations in the water bath, we added tubes into the bath in small groups for each time point and removed all the tubes at the end of the assay. At the conclusion of the assay, we transferred flies into their original vials containing food and held the food vials upside down so that unconscious flies would not become stuck in the food. The next day, we recorded the number of survivors or the number dead within each vial (whichever number was smaller). Flies that exhibited the ability to stand stably and walk were considered to be alive, while flies that were immobile, or flies that exhibited spastic motions such as twitching but were not stable in their movements and stance, were considered dead following similar definitions of Czajka and Lee (1990) and Gerken et al. (2015).

**Figure 2:**
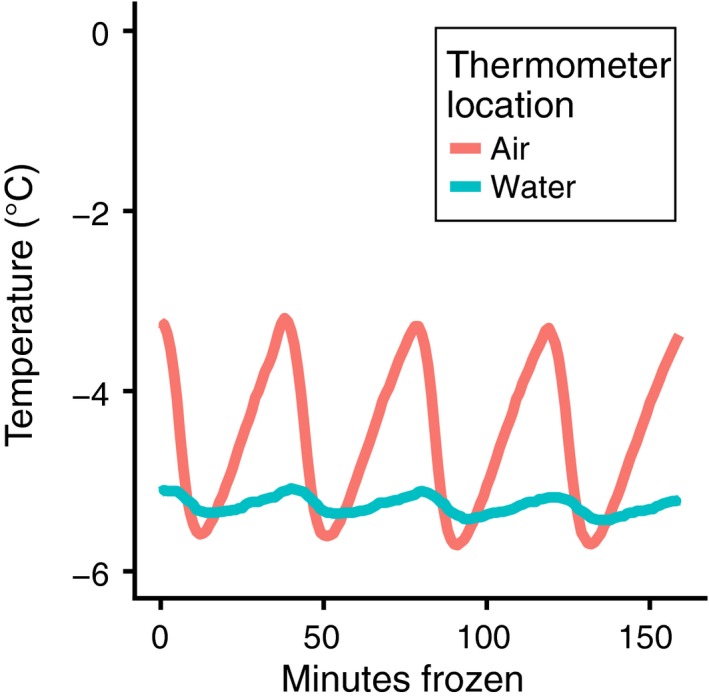
A saltwater bath provides a stable thermal environment during the freeze assay. Temperature probes exposed to air inside the freezer recorded temperature fluctuations ranging from approximately −3.3°C to −5.7°C over the course of the freeze assay. Temperature probes submerged in a saltwater bath inside the freezer recorded temperature fluctuations ranging from approximately −5.1°C to −5.4°C

Due to the high level of thermal sensitivity involved in *D. melanogaster's* cold tolerance (Czajka & Lee, 1990), we designed the freeze assay with the goal of creating the most consistent thermal environment possible both during and between freeze assays. We logged air and water temperature with EL‐WiFi‐DTP+ (dataq.com) temperature probes and the manufacturer's EasyLog software. By placing the Falcon tubes that contained the flies inside a saltwater bath inside the freezer, rather than in the freezer directly, we reduced temperature fluctuations experienced by the flies. Air temperature in the freezer fluctuated by several degrees Celsius, whereas temperature in the saltwater bath varied on the scale of tenths of a degree (Figure 2).

### 2.4 Seasonal assay

Field studies were conducted at Morven Farms in Charlottesville, VA (37°58′02.9″N, 78°28′26.4″W). We established outdoor cages (2 m × 2 m × 2 m) surrounding peach trees and initiated each population with approximately 3,000 flies from our indoor hybrid swarm populations. Cages 1–6 were started on 2 June 2018, and cages 7–11 were started on 11 September 2018. Cages 1–6 were fed with bananas and apples (approximately 3.6 kg of each added to the cages weekly until November, when feeding was done biweekly) and were initially inoculated with live yeast. Cages 7–11 were fed weekly with 2.5 L of cornmeal–molasses fly food, the same food that fed the indoor cages. We collected G0 flies from the outdoor cages using nets or aspirators. Note that although these flies are actually advanced generations of the outbred populations, we refer to them as G0s for the given collection point. On the same day, we collected G0 flies from the indoor cages. We subjected males from the indoor and outdoor cages to the cold hardening and freeze assays as described above. We used only males because female *D. melanogaster* cannot be readily distinguished from another common inhabitant of orchards, *D. simulans*, based on external morphology (Markow & O'Grady, 2006). Our outdoor cages were not completely impenetrable to other insect species, and *D. simulans* males were occasionally found in our collections.

In the laboratory, we established 25 isofemale lines from each cage in vials. We screened the offspring of the isofemale lines for presence of *D. simulans* and discarded these lines (on average, fewer than 10/150 lines per collection time point). We combined all *D. melanogaster* G1s together to collect males for the freeze assay and to establish G2s. G1 males collected from the isofemale lines were cold hardened, frozen, and assayed for survival. The G1 flies were propagated in bottles, and the G2 males were cold hardened, frozen, and assayed for survival.

Heavy rain limited the 7/24/18 collection; we obtained sufficient G0 females to establish lines but not enough males to generate a freeze survival curve. All G1 isofemale lines from the 11/7/18 and 11/21/18 collections were lost in an incubator failure, so we were unable to assay the offspring of these collections. We collected again on 11/30/18 to replace these samples but did not perform a freeze assay on the 11/30/18 G0s. We downloaded weather data from a station located at Carter Mountain, which is approximately 2 km from our field site, to obtain information on average daily temperatures during our experiments (https://www.wunderground.com/dashboard/pws/KVACHARL73).

### 2.5 Nutrition and antimicrobial assays

We made standard cornmeal–molasses fly food in a small batch using a hot plate and added standard amounts of Tegosept and propionic acid to half the batch (Table 1). We used Agricor Fine Yellow Cornmeal and Golden Barrel Blackstrap Molasses in our cornmeal–molasses fly food. In a separate batch of food, we substituted the cornmeal and molasses by volume with pureed bananas (Table 1). We added standard concentrations of Tegosept and propionic acid to half of this batch as well. We allowed flies from each hybrid swarm cage to lay eggs in bottles of the different food types. After these eggs matured into adult flies, we collected males from all treatments and placed them in vials containing standard cornmeal–molasses fly food, cold hardened them, and subjected them to the freeze assay. Therefore, the developmental nutritional environment varied, but adult nutrition during the cold hardening period was identical across all assays.

**Table 1:** Ingredient list and nutritional analysis of laboratory fly food

Banana food ingredients	Cornmeal–molasses food ingredients
315 ml water	315 ml water
3.33 g agar	3.33 g agar
45 ml banana, pureed	22.5 ml cornmeal
	22.5 ml molasses
9.27 g yeast	9.27 g yeast
**Added to ½ batch**	**Added to ½ batch**
2.53 ml 10% Tegosept	2.53 ml 10% Tegosept
0.9 ml Propionic acid	0.9 ml Propionic acid

Nutrition from banana^a^	Nutrition from cornmeal^b^ and molasses^c^
0 g fat	0.27 g fat (cornmeal) + 0 g fat (molasses) = 0.27 g fat
6.3 g sugar	0.25 g sugar (cornmeal) + 15 g sugar (molasses) = 15.25 g sugar

a https://www.fda.gov/food/labelingnutrition/ucm063482.htm.

b https://ndb.nal.usda.gov/ndb/foods/.

c Manufacturer's nutrition label.

### 2.6 Supplemental yeast assay

We collected eggs from indoor hybrid swarm cages in bottles with cornmeal–molasses fly food. We added live bakers' yeast to the surface of half of the bottles and continued to supplement these bottles with yeast throughout the development of the flies. We collected adult males from each treatment and placed them in vials with standard fly food and no live yeast. We cold hardened them for 2 weeks and subjected them to the freeze assay.

### 2.7 Age assay

We collected embryos from the indoor hybrid swarm cages at 2‐week intervals for 6 weeks and reared them to adulthood. We passaged the adult flies to fresh food weekly to prevent eclosion of new adults. We collected adult males once the youngest of the cohorts had eclosed, and we then cold hardened, froze, and measured survival of flies from all three age cohorts in a single assay.

### 2.8 Density assay

We based our density assay on a previous study (Henry, Renault, & Colinet, 2018). We used four density levels: approximately 5 embryos/ml, 40 embryos/ml, 120 embryos/ml, and 300 embryos/ml of fly food. We collected embryos from the indoor cages on cornmeal–molasses fly food plates and counted embryos into vials containing 2 ml of cornmeal–molasses fly food. As higher density vials took longer to develop, we waited to collect adults until every vial had sufficiently eclosed. As a result, the flies in higher density vials were several days younger than the flies in lower density vials at the time of cold hardening. We collected adult males and subjected them to the cold hardening and freeze assay.

### 2.9 Analysis

We analyzed our results using R (version 3.4.2; R Core Team, 2017). All freeze survival curves for the indoor and outdoor cages and their progeny were analyzed using mixed‐effect binomial general linear models using the package *lme4* (Bates, Maechler, Bolker, & Walker, 2015). Cage ID was a random effect, while cage location, hybrid swarm population, and time frozen were fixed effects. We calculated LT50 for each treatment group (collection date, generation, and cage location) by pooling all replicates and using the *dose.p()* function from the *MASS* package (Venables & Ripley, 2002).

All laboratory environmental manipulations were analyzed using binomial general linear models with all factors included as fixed effects. Model results were summarized with the *aov()* function. We used packages *data.table* (Dowle & Srinivasan, 2017), *foreach* (Microsoft & Weston, 2018), *ggplot2* (Wickham, 2009), and *cowplot* (Wilke, 2017) for data manipulation, looping, and graphing. We used *lubridate* (Grolemund & Wickham, 2011) for date conversions. All general linear model results are summarized with an ANOVA.

## 3 RESULTS

### 3.1 Effects of seasonal exposure on post‐cold hardening freeze tolerance

**Figure 3:**
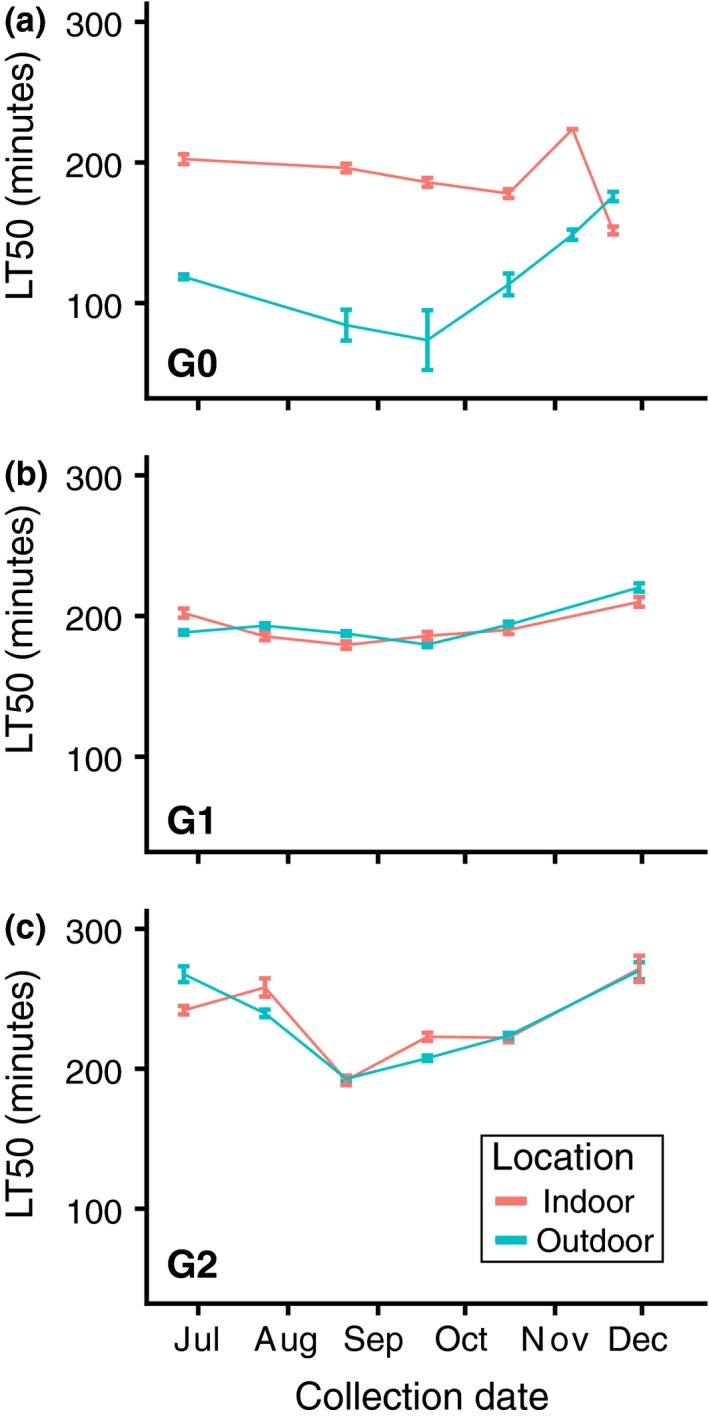
Seasonal plasticity, but not adaptive tracking, in freeze survival following cold hardening. We calculated the LT50, or the time during the freeze assay at which 50% of flies perished, using a general linear model. (a) The G0 generation was collected directly from the indoor or outdoor cages. In the G0 generation, we observed significantly lower LT50s for outdoor flies relative to indoor flies during the summer and fall seasons (Table 2). However, in the late November collection, we observed an increased LT50 for outdoor flies compared with indoor flies (*p* = 5.31 × 10^–7^). (b, c) The G1 and G2 generations were reared in laboratory conditions. We generally did not observe differences between LT50 values for outdoor and indoor flies in the G1 and G2 generations, regardless of collection time (Table 2). Error bars represent standard error of the LT50. Standard error for G0 indoor data from 7 November 2018 is set to zero for clarity (*SE* = 2,927.8 due to incomplete survival curve)

To test the hypothesis that seasonal conditions influence freeze survival following a cold hardening treatment, we collected monthly samples of outbred flies reared in fruit‐fed outdoor mesocosms and cornmeal–molasses‐fed laboratory cages and assessed their cold tolerance following 2 weeks of cold hardening in the laboratory. We used the resulting survival curves to calculate the time required to kill 50% of the flies (hereafter, “LT50”). From June to early November, the LT50 of fruit‐fed, outdoor G0 flies was significantly lower than the LT50 of indoor G0 flies (Figure 3a; Table 2). However, in late November, the outdoor G0 flies had an LT50 greater than that of the indoor G0 flies (Figure 3a; Table 2). We collected outdoor flies on 10 December 2018, but the sample size was insufficient to generate a survival curve. However, the limited data matched the trend from the November collection; after being frozen for 164 min, the survival for the outdoor flies was 84%, while the survival for the indoor flies was 72% (Fisher's exact test, *p* = .39).

**Table 2:** General linear mixed‐effects model comparisons of freeze tolerance of outdoor and indoor flies across collection points and generations

Generation	Collection date	*p*‐value	Direction
G0	6/26/2018	**2.17 × 10^–31^**	Indoor greater
G0	8/21/2018	**4.41 × 10^–23^**	Indoor greater
G0	9/18/2018	**1.40 × 10^–16^**	Indoor greater
G0	10/16/2018	**3.44 × 10^–14^**	Indoor greater
G0	11/7/2018	**7.42 × 10^–14^**	Indoor greater
G0	11/21/2018	**5.31 × 10^–7^**	Outdoor greater
G1	6/26/2018	**1.33 × 10^–4^**	Indoor greater
G1	7/24/2018	.162	–
G1	8/21/2018	.056	–
G1	9/18/2018	.106	–
G1	10/16/2018	.562	–
G1	11/30/2018	.082	–
G2	6/26/2018	.022	–
G2	7/24/2018	.027	–
G2	8/21/2018	.853	–
G2	9/18/2018	**.003**	Indoor greater
G2	10/16/2018	.833	–
G2	11/30/2018	.099	–

Note

Bold text indicates tests that pass Bonferroni correction. Direction indicates which population had greater LT50 (higher freeze tolerance).

**Figure 4:**
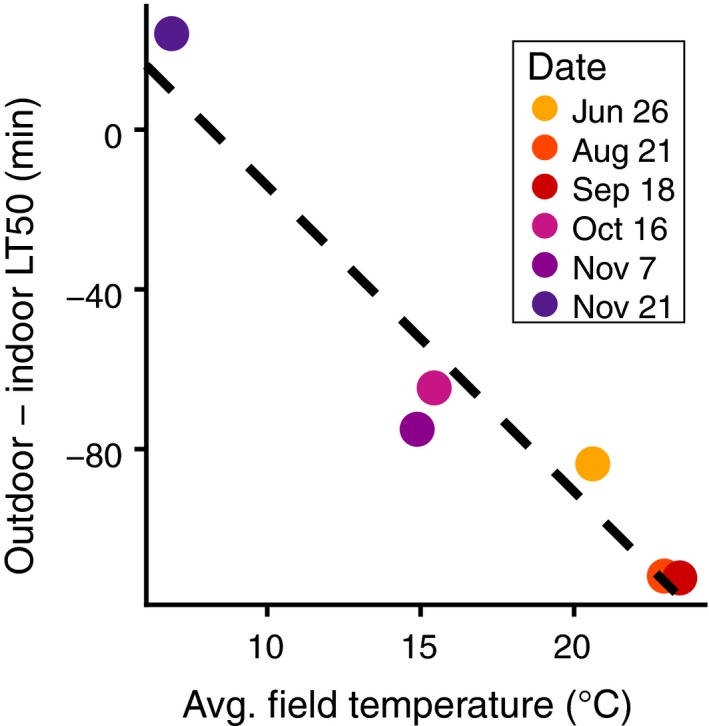
Improved post‐cold hardening freeze survival with decreasing field temperatures. Temperature on day of collection is negatively correlated with the difference in LT50 values for outdoor and indoor G0 flies (linear model; *R* ^2^ = .90, *p* = .002)

In the field experiment, we observed that the effects of the environment prior to cold hardening persisted through the 2‐week cold hardening period. In order to test whether the thermal environment experienced by the flies before cold hardening influenced their post‐cold hardening freeze tolerance, we tested for a relationship between the average temperature on the day of collection and the difference in the post‐cold hardening freeze tolerance of outdoor and indoor G0 flies. We observed a significant negative correlation between the average temperature on the day of collection and the difference in LT50 (Figure 4; linear model; *R* ^2^ = .90, *p* = .0024). Therefore, as temperatures became colder, the cold hardened freeze tolerance increased linearly for the outdoor G0 flies relative to the indoor flies. The regression is also significant when the coldest collection is excluded (*R* ^2^ = .81, *p* = .02), suggesting that prior exposure affects post‐cold hardening freeze tolerance even at moderate to warm temperatures.

To test for transgenerational effects of seasonal exposure on post‐cold hardening freeze tolerance, we compared the laboratory‐reared G1 offspring of flies collected indoors to the laboratory‐reared G1 offspring of flies collected outdoors in a common garden assay. We observed that post‐cold hardening freeze tolerance was generally consistent between the laboratory‐reared offspring of flies collected from indoor and outdoor cages (Figure 3b, Table 2). The similarity between indoor and outdoor G1 flies suggests that the differences in post‐cold hardening freeze tolerance in the G0 flies were not passed on to their offspring. We note that although indoor and outdoor LT50s were significantly different for the 6/26/18 collection (Table 2), we did not observe a consistent difference or pattern in indoor versus outdoor G1 post‐cold hardening freeze tolerances.

We tested for genetic changes in post‐cold hardening freeze tolerance by examining the laboratory‐reared G2 offspring of flies from indoor and outdoor cages. As in the G1s, we also observed little difference in post‐cold hardening freeze tolerance between outdoor and indoor flies (Figure 3c; Table 2). We note that the difference in LT50 between indoor and outdoor G2s was significant for the 9/18/18 collection, but again we did not observe a consistent difference or pattern between the indoor and outdoor G2 post‐cold hardening freeze tolerances. Therefore, our data do not provide evidence of seasonal genetic changes in freeze survival following cold hardening. Although we observed some differences in the overall cold hardened freeze tolerance of G2 flies across collection points (Figure 3c; compare July and August), these seasonal changes occurred in parallel between the indoor and outdoor populations, so we attribute this pattern to experimental artifact and not seasonal evolution. This result emphasizes the invaluable nature of internal controls—in this case, the indoor cages—when conducting seasonal experiments.

Surprisingly, we observed that G2 flies were more freeze tolerant following cold hardening than their G1 parents for some collections (Figure 3b,c, compare July G1s and G2s). Differences in rearing conditions between the generations may have contributed to the differences in post‐cold hardening freeze tolerance: G1 flies were reared in vials, whereas G2 flies were reared in bottles. We investigated whether container type was a potential cause by rearing the 9/18/18 set of G1 flies in both bottles and vials. We did not observe significant differences in post‐cold hardening freeze tolerance resulting from differences in container type (Table 3; *p* = .191), though vial‐reared flies had a slight increase in post‐cold hardening freeze tolerance compared to bottle‐reared flies. We suggest that differences in freeze tolerance between the G1 and G2 generations may have been caused by stochastic differences in rearing conditions in the G2 bottles as compared to the relatively consistent conditions of G1 isofemale lines.

**Table 3:** General linear models of the effects of experimentally manipulated rearing conditions on post‐cold hardening freeze tolerance

Figure	Factor	*df*	Sum sq	Mean sq	*F*	*p*
N/A	Minutes frozen	1	315.19	315.19	519.36	**<2 × 10^–16^**
	Bottle versus vial	1	1.06	1.06	1.75	.19
	Indoor versus outdoor	1	0.20	0.20	0.34	.56
	Cage	6	1.12	0.19	0.31	.93
	Residuals	68	41.27	0.61		
5A	Minutes frozen	1	20.82	20.82	26.04	**1.61 × 10^–4^**
	Indoor versus outdoor	1	21.17	21.17	26.48	**1.49 × 10^–4^**
	Cage	6	1.63	0.27	0.34	.91
	Residuals	14	11.20	0.80		
5B	Minutes frozen	1	114.36	114.36	328.59	**<2 × 10^–16^**
	Indoor versus outdoor	1	0.70	0.70	2.00	.17
	Cage	5	2.19	0.44	1.26	.30
	Residuals	34	11.83	0.35		
6A	Minutes frozen	1	118.40	118.40	132.06	**1.08 × 10^–14^**
	Food substrate	1	51.74	51.74	57.71	**1.78 × 10^–9^**
	Antimicrobials	1	0.00	0.00	0.005	.94
	Cage	1	0.14	0.14	0.16	.69
	Residuals	43	38.55	0.90		
6B	Minutes frozen	1	18.04	18.04	30.58	**5.54 × 10^–4^**
	Supplemental yeast	1	5.31	5.31	9.01	**.017**
	Cage	1	3.18	3.18	5.39	**.049**
	Residuals	8	4.72	0.59		
6C	Minutes frozen	1	31.73	31.73	26.20	**1.82 × 10^–5^**
	Age	2	86.45	43.22	35.70	**1.52 × 10^–8^**
	Cage	1	0.44	0.44	0.367	.55
	Residuals	29	35.12	1.21		
6D	Minutes frozen	1	40.49	40.49	50.37	**8.09 × 10^–6^**
	Density	2	12.34	6.17	7.68	**.0063**
	Cage	1	0.00	0.00	0.00	.99
	Residuals	13	10.45	0.80		

Note

Tests conducted only on time points with complete data.

Bold values indicate *p* < .05.

### 3.2 Plastic effects of nutrition on post‐cold hardening freeze tolerance

**Figure 5:**
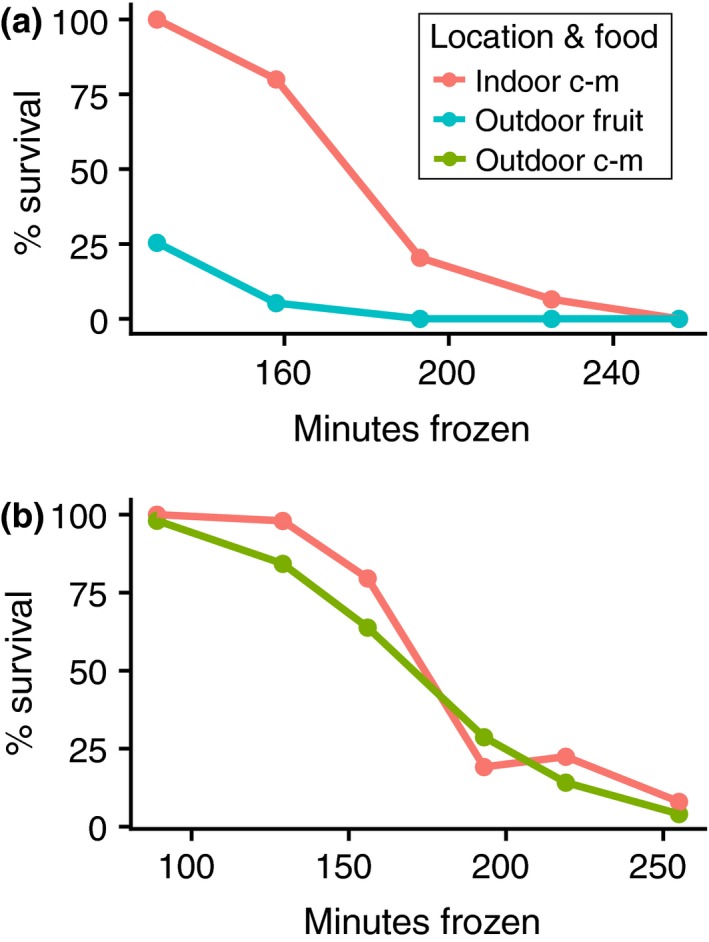
Post‐cold hardening freeze survival varies between outdoor flies fed different substrates. Cold hardened freeze survival curves for G0 flies collected from fruit‐fed cages on 16 October 2018 (a) and cornmeal–molasses (c–m) fed cages 15 October 2018 (b) relative to indoor controls collected on each day. Although outdoor flies from both food treatments experienced comparable thermal conditions, we observed a significant difference in post‐cold hardening freeze tolerance for indoor flies versus outdoor fruit‐fed flies (*p* = 1.49 × 10^–4^, a; Table 3) but not for indoor flies versus outdoor cornmeal–molasses‐fed flies (*p* = .17, b; Table 3). Outdoor survival curves are pooled from six cages in a and five cages in b. Indoor survival curves are pooled from two cages

In addition to experiencing different thermal environments, the indoor and outdoor flies described above consumed different foods prior to cold hardening, which led to the hypothesis that differences in nutritional intake might also affect cold hardening in the seasonal experiments. In October, we collected G0 flies from both the cornmeal–molasses‐fed outdoor cages and the fruit‐fed outdoor cages within 1 day of each other. While the fruit‐fed outdoor flies exhibited a post‐cold hardening freeze tolerance significantly lower than that of the indoor flies (Figure 5a; Table 3; *p* = 1.49 × 10^–4^), the cornmeal–molasses‐fed outdoor flies exhibited comparable freeze tolerance to the indoor flies (Figure 5b; Table 3; *p* = .17). Therefore, flies that experienced nearly identical outdoor thermal regimes greatly differed in post‐cold hardening freeze tolerance depending on their nutritional exposure. Notably, the majority of flies in the cornmeal–molasses‐fed outdoor cages died prior to our next collection at the end of November, despite their possibly enhanced cold hardening ability. The mass mortality of these cages may have been due to the absence of thermal refugia (rotting fruit) during subfreezing temperatures in late fall.

**Figure 6:**
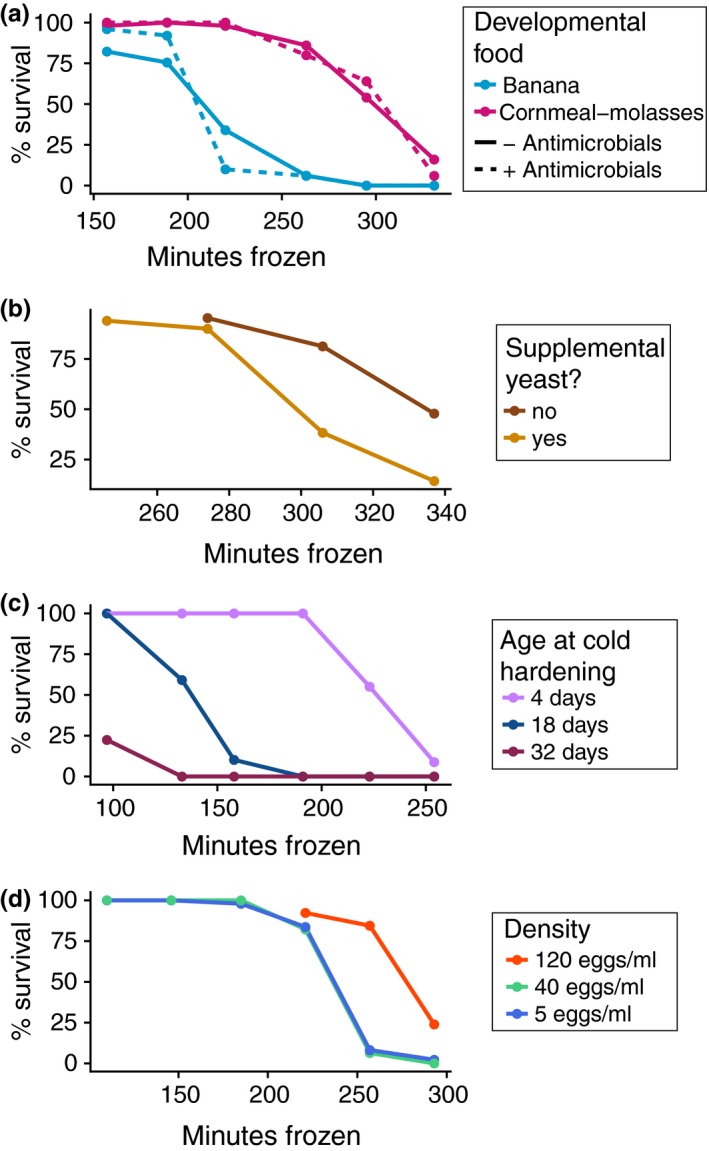
Life history and nutrition influence post‐cold hardening freeze survival. (a) Post‐cold hardening freeze survival curves for indoor flies reared on banana‐based or cornmeal–molasses food, with or without antimicrobials added. Freeze survival is higher for cold‐hardened flies reared on cornmeal–molasses food compared with cold‐hardened flies reared on banana‐based food (Table 3; *p* = 1.78 × 10^–9^). No effect of antimicrobials was observed (*p* = .95). (b) Post‐cold hardening freeze survival curves for flies reared with or without supplemental yeast. Flies supplemented with yeast prior to cold hardening exhibited a slightly decreased freeze survival (Table 3; *p* = .017). (c) Post‐cold hardening freeze survival curves of indoor flies of varying ages. Older flies exhibited lower freeze survival, while younger flies exhibited a higher freeze survival (Table 3; *p* = 1.52 × 10^–8^). (d) Post‐cold hardening freeze survival of flies reared under varying larval density conditions. Flies developed in low‐ and mid‐density conditions (5 eggs/ml and 40 eggs/ml) had similar freeze survival following cold hardening. Flies developed in high density conditions (120 eggs/ml) exhibited improved freeze survival relative to low‐ and mid‐density flies (Table 3; *p = *.0063). All survival curves are pooled from two replicates: one from cage A and one from cage B

We tested several variables that could explain the differences in cold hardening in G0 flies reared on different foods under similar thermal conditions. First, we compared the post‐cold hardening freeze tolerance of indoor flies reared on either a fruit substrate (banana‐based) or the cornmeal–molasses substrate. We observed that flies reared on banana‐based food exhibited a decreased post‐cold hardening freeze tolerance relative to flies reared on cornmeal–molasses food (Figure 6a; Table 3; *p* = 1.78 × 10^–9^). We also tested whether adding antimicrobials influenced cold hardening, since the cornmeal–molasses food contained Tegosept and propionic acid while the rotting fruit did not. We did not observe an effect of antimicrobial presence on post‐cold hardening freeze tolerance (Figure 6a, Table 3; *p* = .95). Therefore, we suggest the cornmeal–molasses diet improved the indoor G0 post‐cold hardening freeze tolerance relative to outdoor flies during summer months (Figure 3a) and also improved the post‐cold hardening freeze tolerance of outdoor flies fed cornmeal–molasses food (Figure 5).

We also hypothesized that yeast availability could contribute to differences in cold hardening. Specifically, the cornmeal–molasses fly food contained yeast as an ingredient, while the cages with apples and bananas relied on yeast growth following an inoculation plus any naturally occurring yeast. We observed a slight decrease in post‐cold hardening freeze tolerance for flies supplemented with extra yeast (Figure 6b; Table 3; *p* = .017). Though we cannot directly quantify yeast availability in the outdoor cages, seasonal variation in yeast growth may have had a minor influence on post‐cold hardening freeze tolerance in the flies in the fruit‐fed outdoor cages.

### 3.3 Plastic effects of life history on post‐cold hardening freeze tolerance

The indoor flies were maintained on a 2‐week generation cycle, while the outdoor flies were able to breed in overlapping generations, likely resulting in a more complex age structure in the outdoor cages. To determine whether age differences could explain the indoor–outdoor differences, we tested the post‐cold hardening freeze tolerance of laboratory‐reared flies of various ages. We observed that younger flies had greater post‐cold hardening freeze tolerances than older flies (Figure 6c; Table 3; *p* = 1.52 × 10^–8^). Although differences in age structure could potentially explain the lower post‐cold hardening freeze tolerance exhibited by fruit‐fed outdoor G0 flies as compared to indoor G0 flies, we expect that age structure between the fruit‐fed and cornmeal–molasses‐fed outdoor cages (shown in Figure 5) should be similar. If age structure alone was causing the outdoor G0 flies to have a lower post‐cold hardening freeze tolerance in the summer and fall, we would expect the cornmeal–molasses‐fed outdoor cages to also have decreased post‐cold hardening freeze tolerance, which was not the case.

Density is a final possibly influential factor in the indoor–outdoor cold hardening differences, since different food substrates and different age structures could lead to different larval densities. We reared larvae at varying densities and observed an increase in post‐cold hardening freeze tolerance at relatively high densities only (Figure 6d; Table 3; *p* = .0063). We suggest that differences in density may have contributed to the observed post‐cold hardening freeze tolerance differences in the outdoor and indoor cages; however, factors such as thermal environment and nutrition are more compelling contributing factors given our experimental results.

## 4 DISCUSSION

Many phenotypes undergo seasonal fluctuations in response to the varying demands of temperate environments. Some organisms exhibit phenotypic plasticity, meaning that an environmental stimulus induces a change in the phenotype. Some populations adaptively track, meaning that some genotypes are more favorable in a given season, and therefore, individuals having those genotypes will be more abundant during that season. In our study, we asked whether the post‐cold hardening freeze tolerance in *D. melanogaster* varies seasonally and whether such variation is a product of plasticity or adaptive tracking. We found that post‐cold hardening freeze tolerance increases as outdoor temperature decreases at the onset of winter. We also determined that, while post‐cold hardening freeze tolerance is highly plastic, the trait does not undergo seasonal evolution. Therefore, we conclude that seasonal fluctuations in post‐cold hardening freeze tolerance are governed by environmental and developmental variables rather than adaptive tracking.

### 4.1 Post‐cold hardening freeze tolerance varies seasonally

Previous studies have demonstrated that cold hardening occurs under natural conditions in *D. melanogaster* using field studies (Kelty, 2007; Overgaard & Sørensen, 2008). These studies placed flies outdoors and then measured their cold tolerance following the natural cold hardening treatment. In our work, we further exposed flies to a consistent cold hardening treatment after they were exposed to field conditions. This experimental design allowed us to elucidate the effects of field exposure that persisted through a consistent, controlled cold hardening regime. We found that the onset of winter conditions correlated with an increased post‐cold hardening freeze tolerance for outdoor flies (Figure 3a, Figure 4). Previous data have shown that the cold tolerance of flies kept outdoors for several hours or days correlates negatively with outdoor temperatures (Overgaard & Sørensen, 2008). We have demonstrated that the effect of field conditioning either persists through 2 weeks of cold hardening or modulates the ability to cold harden in laboratory conditions.

We cannot be certain of the exact influence of field exposure on our results because limited population sizes prevented us from testing the basal cold tolerance of each seasonal collection in addition to the cold hardened freeze tolerance. On one hand, winter conditions prior to the laboratory cold hardening treatment could simply serve to extend the cold hardening period, producing a stronger cold hardening response. On the other hand, winter conditions may induce a plastic change in the *ability* to cold harden, thereby enhancing the cold hardening that occurred during the laboratory cold hardening treatment. The former seems more plausible given evidence that longer cold hardening periods result in a greater increase in cold tolerance (Czajka & Lee, 1990) and repeated exposure to cold has an additive effect on cold tolerance (Kelty & Lee, 2001). However, a different study found that flies collected during different seasons expressed similar survival rates to acute cold stress without cold hardening (Noh, Everman, Berger, & Morgan, 2017). If the baseline cold tolerance of flies is indeed constant across the seasons, then we may conclude that colder temperatures prior to cold hardening do enhance the ability to cold harden.

### 4.2 Post‐cold hardening freeze tolerance does not evolve over seasons

Two possible reasons could explain why we did not observe adaptive tracking of post‐cold hardening freeze tolerance (Figure 3b,c; Table 2). One possible explanation is that our outbred populations of *D. melanogaster* carried limited heritable variation in cold hardening. However, two studies have demonstrated heritable variation in this trait in flies from North Carolina, a population that was included in our hybrid swarms (Gerken et al., 2015, 2018). Thus, we suggest that the absence of adaptive tracking in cold hardening is not due to a lack of genetic variation for this trait within the experimental population.

A second possibility is that post‐cold hardening freeze tolerance is not subject to local adaptation over space and time. Several lines of evidence from previous work are consistent with this model. Field studies have tested for evidence of natural selection in cold hardening in wild populations of *D. melanogaster*. Flies native to tropical or temperate regions exhibit a similar capacity to cold harden despite differences in basal cold tolerance, suggesting that cold hardening undergoes minimal evolution across latitudinal clines (Hoffmann & Watson, 1993). Similarly, flies native to temperate or tropical climates also display comparable overwintering survival in a common environment (Mitrovski & Hoffmann, 2001), suggesting that their cold acclimation ability may be similar. Finally, wild‐caught temperate flies and their recent descendants have been shown to exhibit comparable cold hardening ability to laboratory flies, implying that experiencing natural conditions did not select for changes in cold hardening (Kelty, 2007). Given these results, the lack of evolution of post‐cold hardening freeze tolerance in the outdoor cages is not surprising.

Although we did not observe adaptive tracking in cold hardening, this absence of seasonal evolution is not shared by all cold‐related traits. For example, fly populations exposed to seasonal environmental conditions not only exhibit adaptive tracking with respect to chill coma recovery, but also express decreased levels of plasticity when chill coma recovery times are shorter and vice versa (Noh et al., 2017). In addition, the timing of winter reproduction varies between flies originating from tropical and temperate regions (Hoffmann, Scott, Partridge, & Hallas, 2003; Mitrovski & Hoffmann, 2001), and seasonal and latitudinal variation occurs in diapause propensity (Schmidt & Conde, 2006; Schmidt, Matzkin, Ippolito, & Eanes, 2005). A study of diapause induction in the same outdoor cages studied here demonstrated evolution of increased propensity for diapause induction in late fall (P. A. Erickson et al., in revision), suggesting that this population did in fact carry heritable variation for overwintering‐related traits. Notably, we observed obvious decreases in the population sizes of our outdoor cages in late fall, perhaps suggesting that only the most cold‐tolerant flies survived. However, since this population reduction did not affect the average post‐cold hardening freeze tolerance in subsequent generations, we suggest that the mortality in the population was random or did not involve heritable differences in cold hardening.

### 4.3 Freeze survival following cold hardening is plastic and modified by a variety of conditions

Post‐cold hardening freeze tolerance varies seasonally; however, we did not observe evidence of adaptive tracking. Combined with correlations between post‐cold hardening freeze tolerance and outdoor temperature (Figure 4), we conclude that the seasonal variation observed was a result of plasticity. However, the thermal environment is likely not the sole stimulus that triggers plastic changes in post‐cold hardening freeze tolerance.

#### 4.3.1 Nutrition partially explains differences in post‐cold hardening freeze tolerance between indoor and outdoor flies

We demonstrated that the post‐cold hardening freeze tolerance of flies reared on banana‐based food is significantly lower than the post‐cold hardening freeze tolerance of flies reared on cornmeal–molasses food (Figure 6a; Table 3). The cornmeal–molasses food contained slightly more fat and more than doubles the sugar of the banana food (Table 1). A number of studies have examined the effects of nutritional profiles, particularly fats and sugars, on cold hardening ability.

The role of fats in cold hardening could be mediated by a number of physiological processes. Cold hardening typically changes the makeup of lipid membranes (Overgaard, Sørensen, Petersen, Loeschcke, & Holmstrup, 2005; Overgaard et al., 2006; but see MacMillan, Guglielmo, & Sinclair, 2009). Additionally, increasing cholesterol consumption during larval development has been shown to enhance both baseline cold tolerance and the cold hardening response (Shreve, Yi, & Lee, 2007). Although there is little difference in fat content between our foods, increasing dietary sugars in flies can result in greater fat storage (Colinet, Larvor, Bical, & Renault, 2013). Furthermore, differences in the specific fats found in each food could affect cold hardening; the availability of dietary fats can impact the process of desaturation or other alterations to lipid composition (Overgaard et al., 2005).

Dietary sugar itself may also have a direct effect on the cold hardening response. Certain sugars, particularly glucose and trehalose, are increased in the fly following cold hardening, and greater increases in sugars correspond with a greater cold hardening response (Overgaard et al., 2007; but see evidence of dietary sugar lowering basal cold tolerance in Colinet, Larvor, et al., 2013). Flies fed increased levels of sugar, and so possessing greater sugar stores, may be able to elicit a greater cold hardening response either directly or via changes in fat storage. Taken together, sugar serves as a potential mediator of the increased post‐cold hardening freeze tolerance in flies fed cornmeal–molasses food as compared to banana food.

Live yeast is another nutritional factor that may have contributed to plastic differences in cold hardening between indoor and outdoor flies. The indoor flies were fed food that was made with yeast as an ingredient, whereas the outdoor cages received yeast from an initial inoculation and then relied on natural growth. We demonstrated that supplementing laboratory‐reared flies with live yeast resulted in a slightly decreased post‐cold hardening freeze tolerance (Figure 6b; Table 3). In contrast, previous work found that supplementation with live yeast increases basal (not cold hardened) cold tolerance (Colinet & Renault, 2014). Our results therefore suggest that dietary yeast may have a different influence on basal cold tolerance as opposed to cold hardened cold tolerance.

The role of yeast in cold hardening may be linked to its role in modulating life‐history tradeoffs. Higher yeast availability correlates with lower starvation tolerance, reduced lifespan, and higher fecundity, suggesting that yeast modulates a tradeoff between somatic maintenance and reproduction (Chippindale et al., 2004; Simmons & Bradley, 1997; Tu & Tatar, 2003). We suggest that feeding flies increased yeast may prompt them to prioritize reproduction over survival, thereby reducing energetic investment in processes related to cold hardening. If the fruit‐fed cages had high levels of yeast growth in the summer that diminished in the late fall, these changes could have contributed to plastic seasonal variation in cold hardening. Our observation that dietary yeast influences post‐cold hardening freeze tolerance is further evidence that cold hardening is a plastic phenotype that responds to nutritional conditions.

#### 4.3.2 Effects of life history traits on post‐cold hardening freeze tolerance

The outdoor and indoor cages likely varied in density and age structure, and these factors could plausibly contribute to the observed differences in post‐cold hardening freeze tolerance. We found that post‐cold hardening freeze tolerance declines with age in laboratory‐reared flies, perhaps suggesting an age‐dependent mechanism (Figure 6c). Cold hardening occurs in larvae, pupae, and adult flies, but adults appear to exhibit the greatest cold hardening ability (Czajka & Lee, 1990). Previous studies have demonstrated that increased age correlates with increased chill coma recovery time and decreased cold tolerance (Colinet, Siaussat, Bozzolan, & Bowler, 2013; David et al., 1998). Taken together, these data suggest that the ability to cold harden increases over the course of the fly's development and eventually tapers off in late adulthood as a result of age‐related decline. Without knowing the specifics of age structure in the outdoor cages, it is difficult to conclude how age may have influenced cold hardening in the field‐collected samples. However, our laboratory data and the work of others suggest that it may have been a factor, and thus, aging serves as another example of the plasticity of cold hardening.

The outdoor cages contained a large volume of fruit, perhaps resulting in lower larval densities relative to the indoor controls. We found that high developmental density results in an increased post‐cold hardening freeze tolerance (Figure 6d). Previous work has shown that high larval density induces increased heat tolerance (Sørensen & Loeschcke, 2001) and cold tolerance (Henry et al., 2018). Notably, larval crowding has also been shown to increase adult fat content (Zwaan, Bijlsma, & Hoekstra, 1991) which, as discussed above, may result in greater cold hardening ability. Therefore, the likely higher densities experienced by cornmeal–molasses‐fed flies in the outdoor cages may have primed them for improved cold hardening relative to fruit‐fed flies that likely experienced lower densities. The impact of larval density on cold hardening, combined with the influences of age and nutrition, point to the highly plastic nature of this trait.

## 5 CONCLUSIONS

Short‐lived organisms in changing environments face two options for survival: plastic physiological responses or adaptive tracking. While *D. melanogaster* exhibits seasonal adaptive tracking for several phenotypes (Behrman et al., 2018, 2015; Schmidt & Conde, 2006), we found no evidence for genetic changes in cold hardening in flies experiencing natural seasonal conditions. Instead, post‐cold hardening freeze tolerance is highly dependent on a variety of environmental and life history conditions. Understanding the use of plasticity versus adaptive tracking is critical for modeling and predicting how organisms will cope with a changing climate and the associated shifts in environment and habitat range (Hoffmann & Sgrò, 2011; Merila & Hendry, 2014; Oostra, Saastamoinen, Zwaan, & Wheat, 2018; Stoks, Geerts, & Meester, 2014). Based on our data, we concur with Ayrinhac et al. (2004) that the factors that influence plasticity may be more important than standing genetic variation for some organisms facing thermal extremes.

## CONFLICT OF INTEREST

None declared.

## AUTHOR CONTRIBUTIONS

AOB, PAE, and HMS generated the experimental design. HMS and PAE carried out experiments. PAE and HMS conducted analysis and plotting. HMS wrote the first draft of the manuscript and HMS, PAE, and AOB edited and finalized the manuscript. HMS, PAE, and AOB procured funding.

## Data Availability

Data and R scripts used for analysis and plotting are available on Data Dryad (https://doi.org/10.5061/dryad.bzkh1894v).
